# From crystal structure prediction to polymorphic behaviour: Monte Carlo threshold mapping of crystal energy landscapes

**DOI:** 10.1039/d5sc08644b

**Published:** 2026-01-12

**Authors:** Pedro Juan-Royo, Graeme M. Day

**Affiliations:** a School of Chemistry and Chemical Engineering, University of Southampton Southampton SO17 1BJ UK g.m.day@soton.ac.uk

## Abstract

Crystal structure prediction has developed into a valuable tool for anticipating the likely crystalline arrangement that a molecule will adopt, with applications in materials discovery and polymorph screening. Although powerful, crystal structure prediction is usually limited to locating the local minima of the crystal energy surface. We demonstrate how, by mapping the energy barriers between structures, applying the Monte Carlo threshold algorithm provides a richer description of the crystal energy landscape which allows us to rationalize the differences in experimental conditions under which different crystal polymorphs are observed. As a demonstration, we apply the method to three polymorphic polycyclic aromatic hydrocarbons, phenanthrene, pyrene, and perylene.

## Abstract 2

The Monte Carlo threshold algorithm is used to explore the crystal structure prediction landscapes of three polycyclic aromatic hydrocarbons. The resulting disconnectivity graphs rationalise the conditions required to obtain their polymorphs.

## Graphical abstract

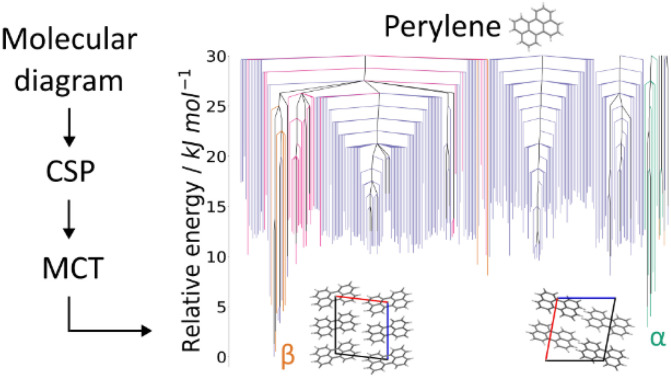

## 1 Introduction

Molecules that crystallize producing different stable crystal structures are said to be polymorphic. Thorough exploration of polymorphic behavior under laboratory conditions is expensive and time-consuming. This is because of the large search space and conditions under which different crystal forms can be produced. In materials discovery experimental programmes, the challenge of exploring experimental variables is coupled with the vast number of molecular structures that might have to be tested to find crystal structures with properties of interest. Computational screening is an option to explore the crystal packing space of a molecular compound and to guide experiments to the molecules of greatest interest and, potentially, to experimental conditions that should lead to particular crystal structures.

Crystal structure prediction (CSP) workflows can be used to explore the crystal packing space of a molecule and identify its thermodynamically stable structures.^1,2^ CSP typically involves the generation of crystal structures that are then energy-minimized to the local minima of the potential energy surface (PES). These structures are then ranked, typically by lattice energy or free energy, to identify possible stable polymorphs. The assumption is that the structures with the lowest energy will be accessible experimentally.^3^

Crystal structure prediction has been used extensively to predict stable crystal structures of small organic molecules,^4^ and benchmarks of the accuracy of the method and its development have been established through a series of blind tests.^5,6^ While CSP is a powerful tool to explore the likely crystal packing of molecules, an enumeration and energy ranking of local minima does not provide a full understanding of the system.^7^ Knowledge of the shape, especially the depth, of the local energy minima, and the energy barriers between them, can provide valuable information relating to the stability of predicted structures.^8^

The Monte Carlo threshold (MCT) algorithm^9,10^ can be used to find the energy barriers between crystal structures in a CSP landscape. The algorithm starts from a local minimum in the PES; in our case, any structure from the CSP landscape could be used. An energy threshold, also called a lid, is set above the energy of the starting point and a Monte Carlo (MC) trajectory is started. The moves are accepted as long as the energy of the structure is not above the lid energy. After a target number of MC moves is reached, the lid energy value is increased, allowing the system to explore higher energy configurations in the PES. The perturbed structures from the MC trajectory can be energy-minimized to obtain local minima of the PES. The lid energy at which a new minimized structure is found serves as an upper bound of the energy barrier between it and the starting structure.

**Fig. 1:**
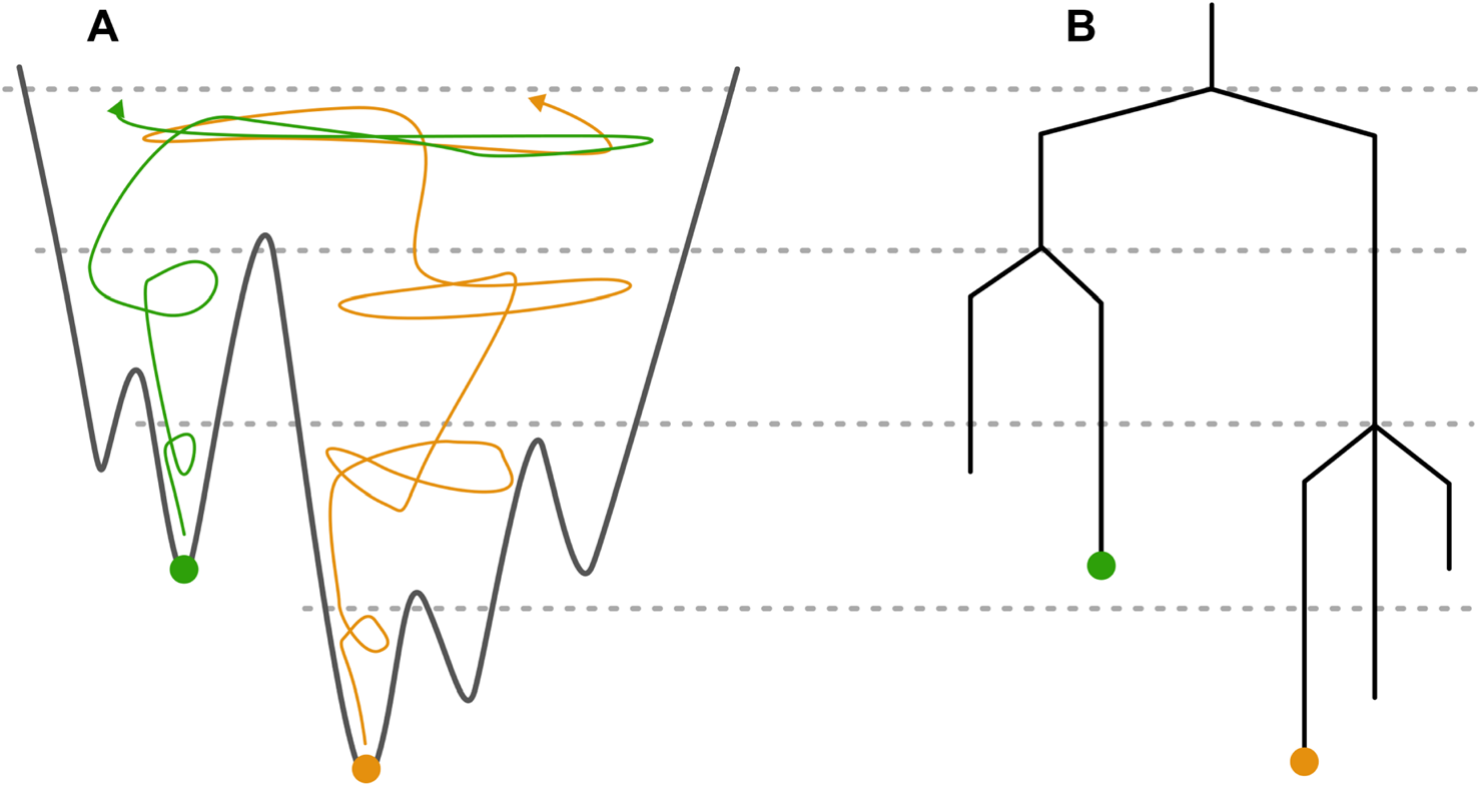
A diagram of a PES (A) and its representation as a disconnectivity graph (B). The dashed lines represent the energy lids, and two example MC trajectories starting from different minima are shown in orange and green. At the highest lid sampled in the diagram, both trajectories merge as they can both minimize to the same structures. It must be noted that exploration of the PES with a MC trajectory is a stochastic process and although two trajectories can sample the same space they might not overlap and in doing so the disconnectivity graph would show two distinct basins, one for each trajectory.

Multiple trajectories starting from different local minima can be merged if the same energy-minimized structures are found between them. Above the lid energy where a matching structure between trajectories is found, both trajectories are exploring the same space of the PES, as shown schematically in Fig. 1A. By starting trajectories from all low energy structures of the PES and incrementing the lid until reaching sufficiently high energy, the MCT algorithm can provide information on all energy barriers separating basins on the PES. This would provide a view of the overall topology of the crystal energy landscape.

The energy barriers and depth of the local minima can be visualised using a disconnectivity graph,^11,12^ which reduces the high dimensionality of the PES into a tree-like structure which can be visualized, and information about energy barriers between structures easily extracted. An example disconnectivity graph is shown in Fig. 1B.

The use of the MCT algorithm for estimating the energy barriers between crystal structures was recently adapted by our group for application to molecular crystals.^8,13^ In our preliminary studies with this method, we used large energy lid increases, of 5 kJ mol^−1^ and 2.5 kJ mol^−1^, and either examined an energy window very close to the global energy minimum or used only the observed crystal structures as starting points for MC trajectories. These limitations mean that the resolution of the barrier heights was poor and a global picture of the crystal landscape could not be obtained.

In this study, we employ CSP and the MCT algorithm together to develop energy landscape maps, and investigate how the landscape structure relates to observed polymorphic behaviour. The approach is applied to three polycyclic aromatic hydrocarbon (PAH) molecules: phenanthrene, pyrene, and perylene. PAHs are molecules containing two or more fused benzene rings consisting only of carbon and hydrogen. Although they are major pollutants and have carcinogenic effects,^14^ the extended conjugation of the π-ring structures makes them appealing for use in organic electronics applications.^15^ The electronic properties of these materials are influenced by many factors, such as crystal packing.^16,17^ Due to their generally planar molecular conformation, PAHs tend to crystallize in a set of defined packing motifs: herringbone (H), sandwich–herringbone (SH), beta (β) and gamma (γ).^18^ The packing that the molecule adopts when in crystal form can depend on the processing conditions, such as temperature,^19^ pressure, and solvents.

In addition to comparing the energy landscapes of the molecules, we investigate the impact of the choice of potential energy model by repeating the CSP and MCT analysis with three different atom–atom potential energy models: FIT,^20^ PAHAP,^21^ and isoPAHAP.^22^ FIT is a transferable potential energy model for crystal structure modelling, and is therefore parametrized from a variety of molecular chemistries. PAHAP is an anisotropic atom–atom potential parametrized exclusively with data of PAHs dimers, and isoPAHAP is an isotropic atom–atom potential derived from PAHAP. All three model potentials were combined with atomic multipole electrostatics.

To ensure that we achieve good energy barrier resolution, we employ a multistage sampling scheme (described in more detail in SI Section S1.2), and to have as complete a coverage of the crystal PES as possible, we use a large set of predicted structures in the CSP landscape as initial points for the MC trajectories.

## 2 Methods

### 2.1 Crystal structure prediction

The structure generation stage was carried out using our GLEE (Global Lattice Energy Explorer)^23^ program, as implemented in mol-CSPy.^24^ Crystal structures were generated in the top 10 most common molecular organic space groups (SGs) with *Z*′ = 1: *Pbca*, *P*2_1_/*c*, *C*2/*c*, *P*2_1_2_1_2_1_, *P*1̄, *P*2_1_, *Pna*2_1_, *Cc*, *Pca*2_1_ and *C*2. The structure generation search terminated once 10 000 structures were generated and successfully energy minimized for each SG. For the pyrene molecule, an extra crystal generation stage was performed with *Z*′ = 2 in SG *P*1̄ targeting 40 000 structures; this was done to find matching structures to the experimental polymorph IV.^25^ The generated structures were lattice-energy minimized in a 3-stage procedure, described in detail in SI Section S1.1.

The set of generated structures was clustered to remove any duplicates. A first quick clustering stage was performed by comparing their simulated powder X-ray diffraction (pXRD) patterns. This was followed by a slower but more accurate comparison with the COMPACK^26^ algorithm as it is implemented in the Cambridge Structural Database^27^ API.

### 2.2 Monte Carlo threshold algorithm

The MCT trajectories were started from the crystal structures of the CSP landscape that matched the experimental polymorphs of each system, plus an extra 30 of the lowest energy predicted crystal structures selected using a generalized convex hull (GCH) algorithm adapted to molecular crystals.^28^ The GCH algorithm identifies predicted structures that are energetically close to the convex hull defined with respect to the structural features that show greatest variance across the CSP landscape. By defining these features from a principal component analysis of a similarity kernel, the GCH does not depend on user defined features to generate the convex hull. By selecting structures close to the GCH, we generate starting pools of crystal structures that spread across a large space of these identified features; this provides a maximally diverse set of low energy starting points. By combining the GCH selection of structures with MCT, we aim to generate a global view of the PES. It must be noted that the crystal structures selected by the GCH algorithm are not equivalent between the three potential energy models. Thus, the similarities that we see in the resulting disconnectivity graphs between potential models (see below) suggest that the global features of the landscape are insensitive to the exact selection of starting structures.

Selected structures were expanded to *P*1 Niggli-reduced cells in order to remove any symmetry constraints on the MC sampling and avoid running the algorithm in stretched cells that can arise in the CSP structure generation step.

Whenever possible, the MCT trajectories were run in unit cells with four molecules. Any supercells that had to be generated were created by repeatedly doubling the original cell along the shortest cell axis. Some predicted crystal structures have unit cells with eight molecules, in which case sampling was performed in the unit cell and twice as much MC sampling was performed than in the cells with four molecules. Running most MCT trajectories in unit cells with four molecules is a choice made to balance out the computational cost and accuracy of the calculations. Larger cells would allow finding lower energy barriers, where small coordinated displacements of many molecules result in the system overcoming the energy barrier between local minima. Larger simulation cells would also be required to adequately model transitions that occur by nucleation and growth, which is common in polymorph transitions for molecular crystals.^29^ However, using larger simulation cells would require much more sampling in each energy lid, which is beyond current capabilities with reasonable computing resources.

A total of three independent MCT trajectories were run from each structure, each one with an increasing amount of sampling of the lower energy regions of the PES. The three sampling schemes, S1, S2, and S3, are shown in Table 1. S1 and S2 are used to sample up to high energies above the starting point to ensure connections between all crystal structures are found. S3 ensures a very thorough sampling of the energy regions near the starting points, allowing us to find the lowest-energy connections possible. The accepted MC steps in the trajectories were lattice-energy minimized following a similar 3-stage procedure to the one used with CSP structures, described in detail in SI Section S1.1. Differently to the CSP energy minimizations, those in the MCT algorithm were done in the *P*1 space group, *i.e.* with no space group constraints. This results in the exploration of a crystal PES with many more local minima than in the CSP search. This is done so that the lowest energy transitions between CSP crystal structures in different space groups can be found.^13^

**Table 1:** Description of the three sampling schemes used for the MC trajectories of the treshold algorithm. S1 and S2 reach the same energy-lid above the starting structure, but S2 has twice the number of MC steps. S3 does not reach as high an energy-lid above the starting point, but it is used to thoroughly explore the low energy region of the PES to find the lowest energy connections possible

Sampling scheme	Total MC moves	MC moves per lid	Lid energy increases (kJ^−1^ mol^−1^)	Max. lid energy (kJ^−1^ mol^−1^)
S1	13 000	1000	5.00	65.00
S2	26 000	1000	2.50	65.00
S3	34 000	2000	1.25	21.25

Connections between MCT trajectories were found by clustering the structures using simulated pXRD patterns. From these connections, the disconnectivity graphs could be constructed.

### 2.3 Potential energy models

Three different potential energy models were employed when energy-minimizing the CSP and MCT generated structures and calculating single-point energies of the MC perturbations: FIT,^20^ PAHAP^21^ and isoPAHAP^22^ descriptions of repulsion and dispersion interactions, each combined with the same atom-centered multipole model for describing electrostatic interactions. FIT is a transferable potential energy model for organic molecular crystal structure modelling, and is therefore parametrized from a variety of molecular chemistries. PAHAP is an anisotropic atom–atom potential parametrized exclusively with data of PAHs dimers, and isoPAHAP is an isotropic potential derived from it. The functional forms of the three potentials are shown in SI Section S1.3. We use atom-centered multipoles up to hexadecapole to describe the electrostatics with all potentials, effectively turning all potentials to an anisotropic atom–atom form. Calculations were performed using the DMACRYS lattice energy modelling software.^30^

## 3 Results

### 3.1 Experimental polymorphs

**Fig. 2:**
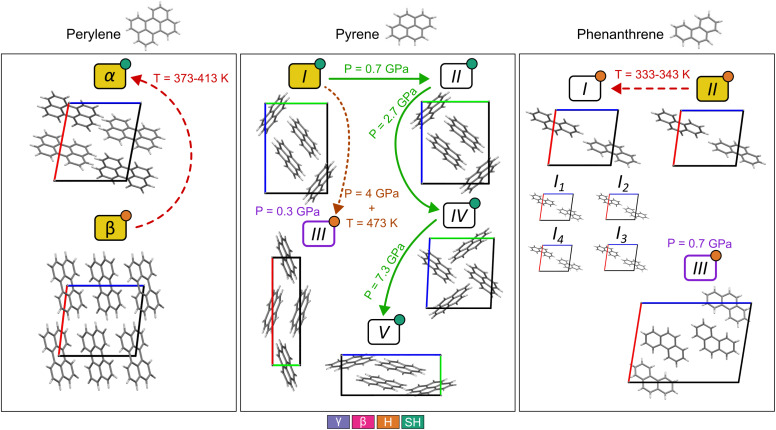
Experimentally observed polymorphs of the three molecules selected for this study. Those with labels in yellow boxes are the polymorphs that are observed at room temperature and pressure. Green solid arrows indicate transitions between polymorphs by applying pressure. Red dashed arrows indicate a transition by increasing the temperature. Orange dotted arrows indicate a transition observed by applying pressure and increased temperature to the sample. Purple outlined polymorphs are obtained by recrystallizing the molecules from a dichloromethane solution under pressure. The packing motif of each crystal is indicated as a coloured circle at the top right corner of each polymorph.

The molecules chosen for this study exhibit polymorphism, summarized schematically in Fig. 2. Perylene has two polymorphs: *α*,^31^ with the SH packing, and *β*,^32^ with a H packing. Both polymorphs are observed at ambient pressure and temperature. An irreversible transformation is observed from *β* to *α* when temperature is increased between 373 K and 413 K.^33^

Pyrene has five experimental polymorphs: I,^34^ II,^25,35^ III,^36^ IV,^25^ and V.^25^ All polymorphs show the SH packing except III, which can be classified as H. I is the ambient pressure polymorph, which undergoes a transformation to II at low temperature and at pressures above 0.7 GPa and below 2.7 GPa. Further compression of II transforms it into IV, which is observed between 2.7 GPa and 7.3 GPa. Beyond 7.3 GPa, V is observed. III was first obtained by recrystallization from a dichloromethane solution under a pressure of 0.3 GPa and high temperature, and it redissolved once pressure was removed.^36^ Prior to the discovery of polymorphs IV and V, Sun and coworkers^37^ observed a transformation of a sample of polymorph I at ∼0.3 GPa by vibrational spectroscopy, which was interpreted as a transition to III based on a reduction in the number of bands in the Raman spectra. We note that the symmetry of forms III and IV are both consistent with the number of Raman bands observed by Sun. Later, Zhou and coworkers demonstrated that I transforms to a mixture of III and IV if the sample is heated to 473 K when pressurized, interpreting this as meaning that the transformation from I to III must overcome a large energy barrier.^25^

Phenanthrene has three experimental polymorphs: I,^38^ II,^39^ and III.^40^ II is the ambient temperature and pressure polymorph. I has the same packing as II but with rotational disorder, observed at temperatures above 339 K. To account for this disorder, four crystal structure models were created with different combinations of molecular orientation in the unit cell, referred to as I_1_ to I_4_. I_1_ is equivalent to I_4_ due to symmetry, and I_2_ and I_3_ are also symmetrically equivalent. More information on how the disordered crystals were handled is explained in SI Section S1.4. III is a high pressure polymorph obtained by recrystallization of phenanthrene in a dichloromethane solution under a pressure of 0.7 GPa and at high temperature, which was not stable once pressure was removed.^40^ All polymorphs have a H packing motif (Fig. 2).

### 3.2 CSP

**Fig. 3:**
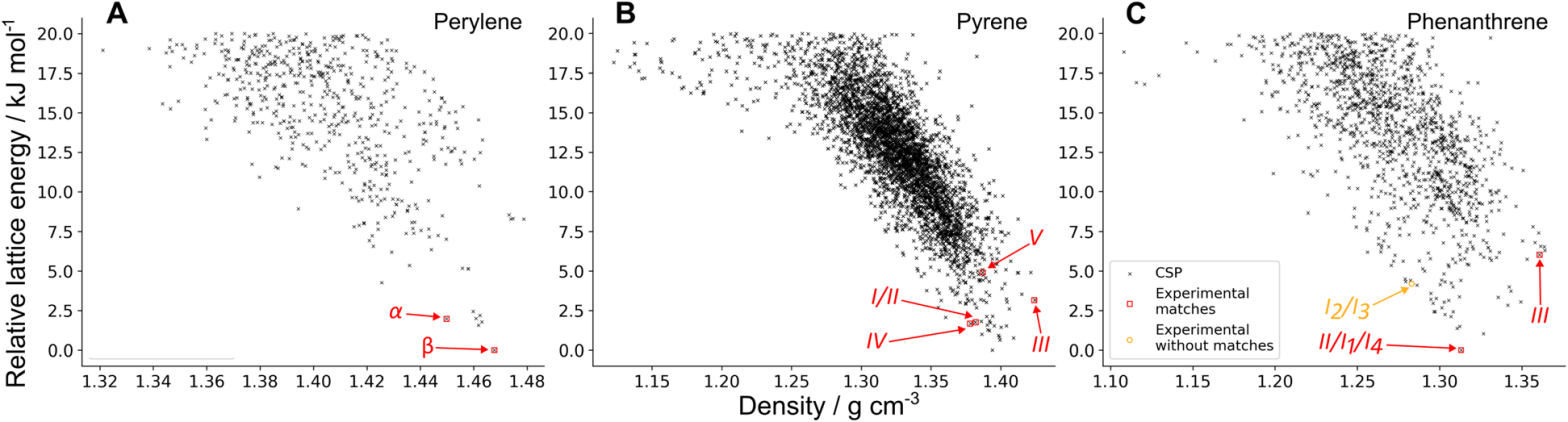
Energy-density plots of the CSP landscapes of the three molecules using the PAHAP potential: perylene (A), pyrene (B), phenanthrene (C). The landscapes have been truncated to show only the structures within 20 kJ mol^−1^ of the global energy minimum for each molecule. Plots of the landscapes of the other potentials are in SI Fig. S3. Black crosses are all the structures generated with the CSP workflow. Red squares indicate the structures that match an experimental crystal, and orange circles are experimental structures that have no match in the landscape.

The CSP landscapes of the three molecules with the PAHAP potential are shown in Fig. 3 as energy-density plots, where each point corresponds to a distinct crystal structure. Data points corresponding to crystal structures that match experimental polymorphs are shown in red, and in orange those experimental polymorphs with no matches in the landscape. The landscapes for the FIT and isoPAHAP potentials can be seen in SI Fig. S3.

The CSP search successfully finds matches for all experimentally observed polymorphs of the three molecules, except two combinations of the disordered polymorph of phenanthrene, I_2_ and I_3_. These two configurations of the disordered polymorph are not sampled in CSP due to the choice of space groups and *Z*′ values used for exploring the crystal packing landscape.

This method provides us with information on the predicted relative energies between experimental polymorphs, their stability with respect to the global energy minimum and the structures and energies of other competing, but as-yet unobserved, crystal forms. This information can be used to rank the experimental matches by their predicted stability relative to each other, shown in Table 2.

**Table 2:** Ranking (top three rows) and lattice energy difference (bottom three rows) in kJ mol^−1^ with respect to global energy minimum of the experimental matches in the CSP landscape. Pyrene polymorphs I and II have the same ranking in the FIT and PAHAP landscapes due to both structures energy-minimizing to the same local minimum in the PES

	Perylene		Pyrene					Phenanthrene		
	*α*	*β*	I	II	III	IV	V	II/I_1_/I_4_	I_2_/I_3_	III
FIT	4	1	42	42	4	55	186	1	32	24
PAHAP	4	1	15	15	33	13	81	1	26	55
isoPAHAP	3	1	12	23	5	24	97	1	19	6
FIT	1.51	0.00	2.77	2.77	0.56	3.04	5.11	0.00	3.78	3.37
PAHAP	1.99	0.00	1.79	1.79	3.17	1.70	4.96	0.00	4.20	6.03
isoPAHAP	2.96	0.00	2.03	2.79	0.99	3.10	6.11	0.00	3.84	1.49

Ranking of the perylene polymorphs is predicted to be: *β* as the most stable, being the global energy minimum with all potentials, followed by *α* with an energy difference of less than 3.0 kJ mol^−1^. These results are the opposite of what experimental data suggests: the observed high temperature transformation from *β* to *α* is irreversible, suggesting that the thermodynamically stable polymorph is *α* and that *β* is metastable at room temperature, and observed for kinetic reasons.

In the case of pyrene, the matches in the landscape have the general trend of higher lattice energies if they are observed experimentally at higher pressures. This is expected, as lattice energy calculations do not include any pressure contributions to the crystal free energy, and higher pressures can stabilise higher energy packings. This trend is broken in the case of the PAHAP potential: the ranking in Table 2 shows that polymorph IV, a high pressure form, is predicted more stable than polymorphs I and II. The FIT and isoPAHAP potentials have similar rankings, in which polymorph III is predicted to be the most stable. It must be noted that isoPAHAP is the only potential in which the polymorph matches for forms I and II do not correspond to the same local minimum in the PES.

The room temperature and pressure polymorph of phenanthrene, form II, is calculated as the global energy minimum for all three potentials (Table 2). The high pressure polymorph recrystallised from dissolution in dichloromethane, form III, appears at higher density and higher in the energy landscape: 3.37 kJ mol^−1^ above form II in FIT, 6.03 kJ mol^−1^ in PAHAP, and 1.49 kJ mol^−1^ in isoPAHAP. Two combinations of the disordered form I, I_1_ and I_4_, match the global energy minimum structure (ranked 1st), while I_2_ and I_3_ are ranked higher in energy: 3.78 kJ mol^−1^ above II in FIT, 4.20 kJ mol^−1^ in PAHAP, and 3.84 kJ mol^−1^ in isoPAHAP.

Comparing the performance of the three potentials, we observe that the ranking of experimental polymorphs is quite consistent across them. However, there are major differences between potentials in the number of local minima in the PESs that they define for all three molecules, shown in Table 3. The PESs defined by the FIT potential has, by a large margin, the largest number of local minima for all three molecules. FIT is a general potential that has been parametrized to reproduce crystal structure data of multiple azahydrocarbons and oxohydrocarbons to work with a large number of molecular chemistries, whereas PAHAP and isoPAHAP are parametrized specifically for this class of molecules, which might result in more realistic, smoother, lattice energy surfaces. Thus, the differences arising due to different paramatrizations (isoPAHAP *vs.* FIT) seem to have more impact on the number of minima for these molecules than the different functional forms of the models (PAHAP *vs.* isoPAHAP).

**Table 3:** Number of crystal structures in the region up to 15.0 kJ mol^−1^ from the global energy minimum of the CSP and MCT (in brackets) landscapes of each molecule and using each of the potential energy models. The larger amount of crystal structures found in the MCT trajectories is mostly due to the fact that the search is performed in SG *P*1

	Perylene	Pyrene	Phenanthrene
FIT	476 (6559)	4238 (120 707)	1203 (89 516)
PAHAP	244 (2475)	2783 (55 340)	737 (51 123)
isoPAHAP	153 (954)	2609 (68 521)	441 (36 140)

From the CSP results, we can rationalize the structures of the room temperature and pressure polymorphs of phenanthrene and perylene as the lowest energy possible crystal packing; this is not the case for pyrene, for which the global energy minimum predicted structure does not correspond to one of the observed polymorphs, although the FIT and isoPAHAP potentials give the correct relationship between polymorphs related by pressure-induced transformations. In general, we lack information that can indicate why we observe both polymorphs of perylene at room temperature and pressure, the appearance of a disordered polymorph in phenanthrene, and why the high pressure forms III of pyrene and phenanthrene require recrystallisation from dissolution in dichloromethane or (for pyrene III) high temperature to induce the transition. We examine whether the MCT algorithm can provide us the missing information.

### 3.3 MCT algorithm

MCT trajectories were started from the crystal structures of the CSP landscape that matched the experimental polymorphs of each system, plus an extra 30 structures to represent the rest of the low energy region of the lattice energy surface. These 30 additional structures were selected from those within 15 kJ mol^−1^ of the global lattice energy minimum using a GCH algorithm^41^ adapted to molecular crystals.^28^

The total number of unique crystal structures that are found with the MCT trajectories is shown in Table 3. The MCT trajectories find many more crystal structures than the CSP search. This is mostly due to running MCT without symmetry constraints, so that the MCT trajectories explore the *P*1 PES, which has many additional local minima of lower symmetry than those found in CSP. The choice of starting local minima for the MCT does not have a large effect on this number, but if we were to start MCT trajectories from more local minima in the landscape the number of unique structures found is expected to continue to increase. The number of starting crystal structures, 30 plus the experimental matches, is chosen as a trade-off between obtaining a general view of the PES and having the computational resources to carry out analysis on all the local minima found during each MCT trajectory.

#### 3.3.1 Perylene

**Fig. 4:**
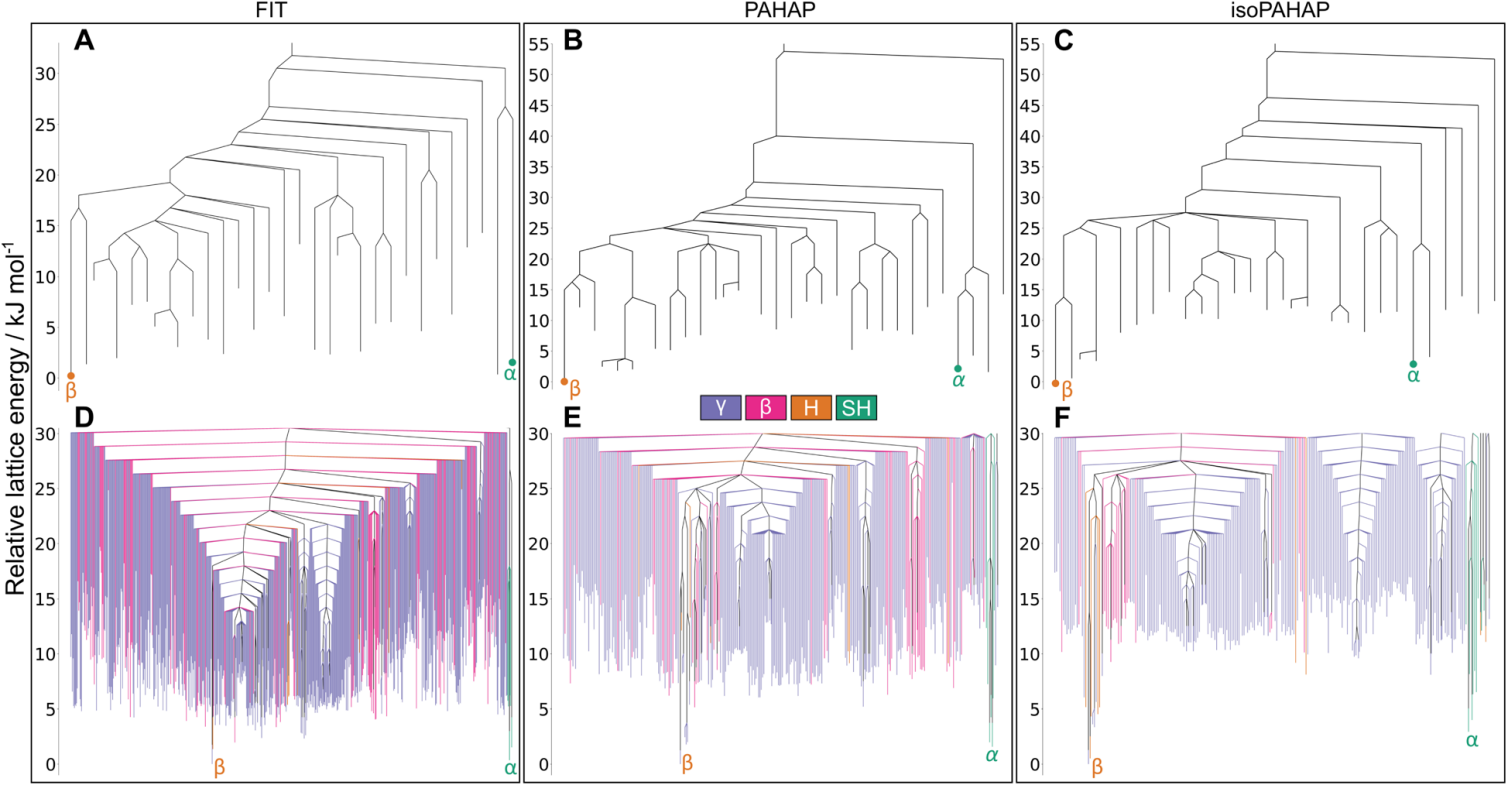
Disconnectivity graphs for the perylene molecular crystal energy landscape. (A–C) Contain the crystal structures from which the MCT trajectories were started, (D–F) show all the local minima found in the MCT trajectories up to a lid 30.0 kJ mol^−1^ above the global energy minimum colour-coded by crystal packing motif. The location of the experimental matches is shown in red. A large energy barrier separates both polymorphs of the molecule (A–C), which could explain why a high temperature is needed to interconvert between them. The basins containing the experimental matches stand out from the rest of the landscape as they have structures with lower lattice energy (D–F), which explains why both polymorphs are observed at room temperature and pressure as the two basins are competing to be the global energy minimum of the landscape.

The disconnectivity graphs of the perylene crystal structure landscape are shown in Fig. 4. Not all the crystal structures found in the MCT trajectories are shown for clarity. The full disconnectivity graphs of all molecules can be seen in SI S5. Fig. 4A–C are the disconnectivity graphs of the crystal structures from which the MCT trajectories were started, where we can easily read the energy barriers between them. Disconnectivity graphs in Fig. 4D–F show a global picture of the low energy region of the landscape, consisting of all the crystal structures found in the MCT trajectories up to a lid energy 30.0 kJ mol^−1^ above the global energy minimum. Each structure is colour-coded by their crystal packing motif.

The structures corresponding to the experimental polymorphs, *α* and *β*, occupy separate energy basins, with calculated energy barriers connecting the structures in the range 30 kJ mol^−1^ to 40 kJ mol^−1^ in all potentials, seen in Fig. 4A–C. A large energy barrier separating *α* and *β* helps explain why both polymorphs appear at room temperature and pressure, as the large energy barrier kinetically stabilizes the non-thermodynamically stable form.

The disconnectivity graphs showing all structures in the low energy region (Fig. 4D–F) of the landscapes also sheds some light on the stability of the experimental polymorphs and the relationship to their packing motifs. The basins containing the experimental forms have different packing motifs than the rest of the landscape. The basin of polymorph *α* is almost exclusively formed of SH structures, while the *β* basin is almost exclusively formed of H structures. These differences are more visible on the PAHAP (Fig. 4E) and isoPAHAP (Fig. 4F) disconnectivity graphs.

Also noticeable is the fact that the energy basins occupied by the experimental crystal structures contain structures with lower lattice energy than the rest of the landscape. This essentially creates two competing low-energy regions of the landscape towards which the system can be driven during crystallization. Therefore, the global structure of the energy landscape provided by the MCT simulations also rationalizes the appearance of both polymorphs under the same conditions and the ability to influence the resulting polymorph through the introduction of templates and additives.^42^

#### 3.3.2 Pyrene

**Fig. 5:**
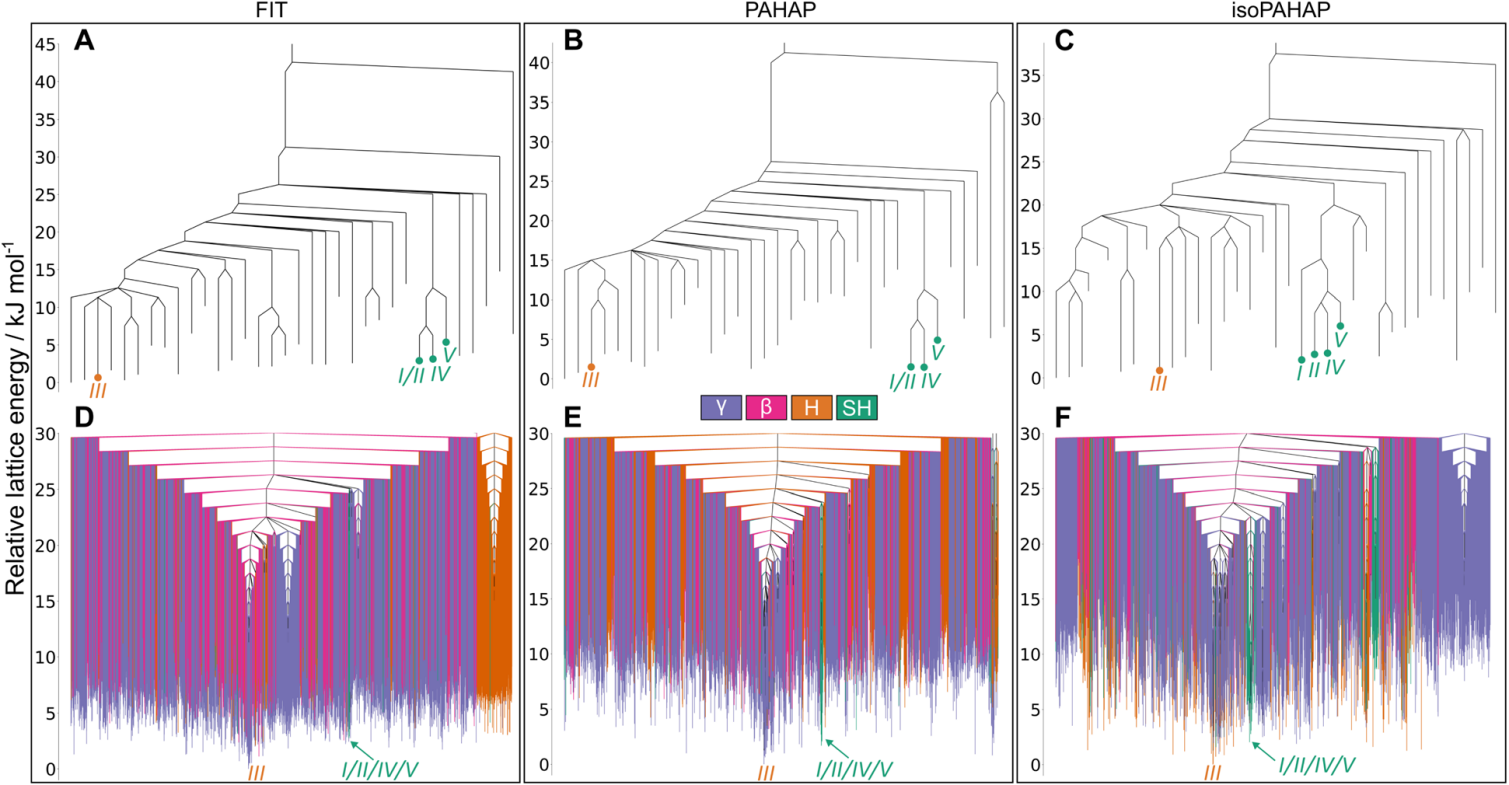
Disconnectivity graphs for the pyrene molecular crystal energy landscape. (A–C) Contain the crystal structures from which the MCT trajectories were started, (D–F) show all the local minima found in the MCT trajectories up to a lid 30.0 kJ mol^−1^ above the global energy minimum colour-coded by crystal packing motif. The location of the experimental matches is shown in red. In (A–C) we can see that there are two basins in the landscape, one containing polymorph III and the other one the rest. This lines up nicely with the experimental conditions under which the polymorphs are observed.

The disconnectivity graphs for the pyrene crystal structure landscape are shown in Fig. 5. In Fig. 5A–C we can see many basins in the landscape, one containing polymorph III, while the rest of the experimentally observed forms (I, II, IV and V) are grouped together in a separate basin. The two basins corresponding to the known polymorphs are separated by a large energy barrier of ∼25 kJ mol^−1^, showing similar behaviour across the three potential energy models. The calculated structure of the energy landscape aligns very nicely with the appearance of pyrene's crystal polymorphs under different conditions. Forms II, IV, and V are obtained by increasingly applying more pressure to a crystal of form I; we find that these transitions to forms IV and V involve low energy barriers. Form III, on the other hand, is obtained either by dissolving the molecule in a solvent and recrystallizing under pressure^36^ or by applying pressure with elevated temperature (473 K). The requirement for high temperature or recrystallization to obtain form III, and the observation that it does not convert to the ambient pressure form upon decompression,^36^ is understandable in terms of the large energy barrier separating this structure from I.

If we focus our attention on the basin with polymorphs I, II, IV, and V, the energy barriers between crystal structures match the increased pressure in which they appear. The energy barrier between polymorphs I and IV is smaller than between I and V. This trend is observed for the FIT (Fig. 5A) and PAHAP (Fig. 5B) potentials. isoPAHAP (Fig. 5C) shows a slightly different energy barrier distribution, in which polymorph I has a large energy barrier with a basin containing polymorphs II, IV, and V. We are confident with our sampling of the low energy regions of the landscape, so this difference in energy barriers must arise from the potential energy model itself.

Looking at the disconnectivity graphs showing the packing motifs of the low energy region of the landscape (Fig. 5D–F), we can focus on the basin containing the I, II, IV, and V polymorphs. The calculated lattice energies of the structures in this basin are not the lowest of the landscape, but this basin stands out as it is exclusively formed of SH packing structures. On the energy landscapes calculated with the FIT (Fig. 5D) and PAHAP (Fig. 5E) potentials, the basin containing I is the only basin with the SH packing; with isoPAHAP (Fig. 5F) there is another basin formed exclusively of SH packing structures at a higher energy lid and with structures of higher lattice energy. Pyrene crystallizes in the SH packing motif at ambient conditions; crystallization drives the system towards this region of the energy landscape formed exclusively of that packing motif.

#### 3.3.3 Phenanthrene

**Fig. 6:**
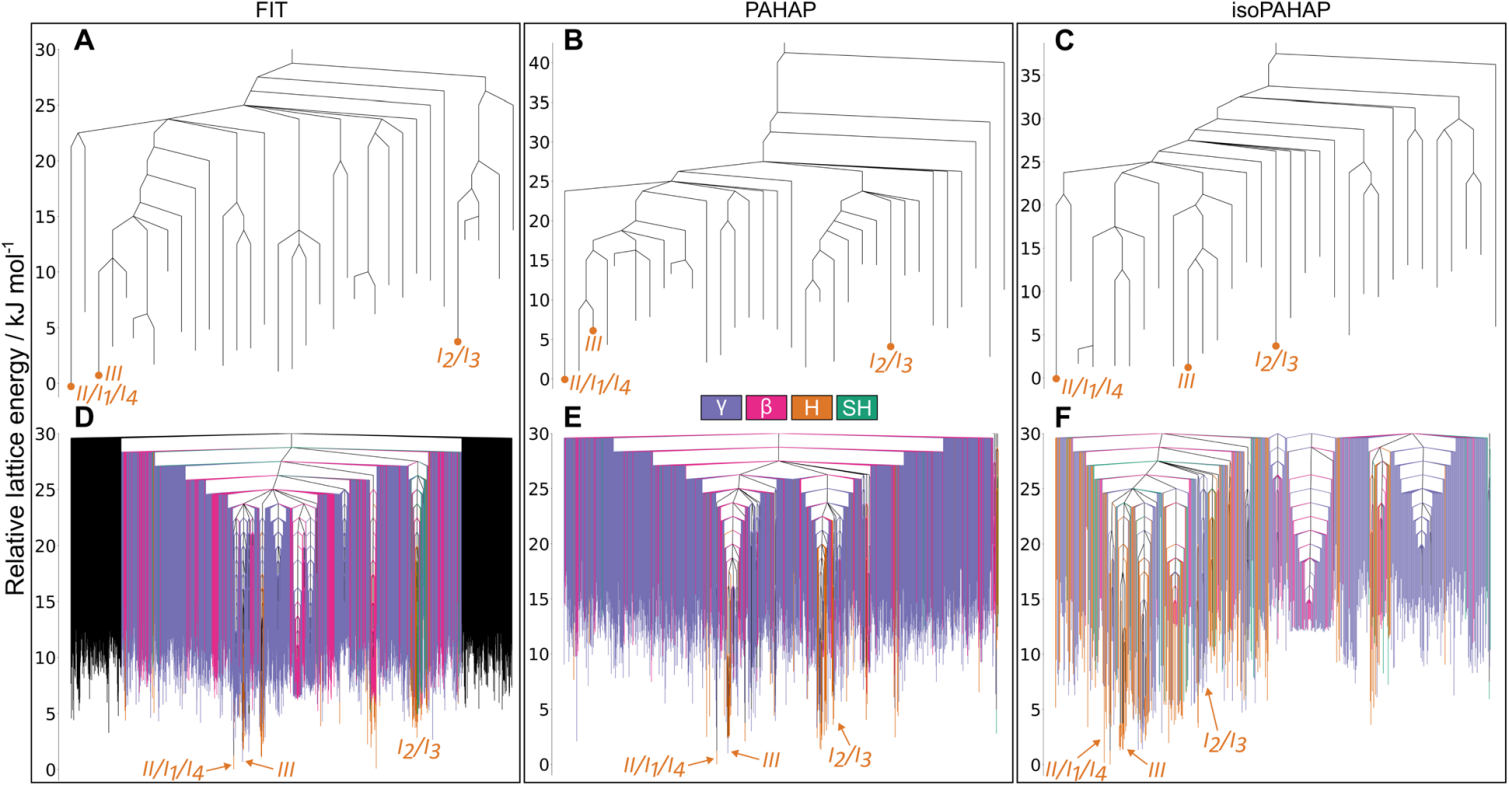
Disconnectivity graphs for the phenanthrene molecular crystal energy landscape. (A–C) Contain the crystal structures from which the MCT trajectories were started, (D–F) show all the local minima found in the MCT trajectories up to a lid 30.0 kJ mol^−1^ above the global energy minimum colour-coded by crystal packing motif. The location of the experimental matches is shown in red. Large energy barriers separate the room temperature form with the high pressure and temperature ones (A–C). (D–F) Show that at low energy lids there are a large number of structures with different kinds of crystal packing, which could indicate that molecules in the crystal can easily shift and rotate. This could explain the appearance of a disordered polymorph.

Disconnectivity graphs of the phenanthrene crystal structure landscape are shown in Fig. 6. In Fig. 6A–C we see that there are large energy barriers between the room temperature and pressure form, II, and the high pressure form III. This high energy barrier explains the need for recrystallisation at high pressure to form III, as for pyrene form III, rather than it being accessible by direct compression. Recrystallisation at high pressure leads the system to a different region of the landscape than crystallisation at room temperature and pressure. The higher density of III (Fig. 4C) and, thus, lower PV contribution to the free energy, explains why high pressure would favour the region of the energy landscape containing III.

The MCT results also reveal a high energy barrier between II and the I_2_/I_3_ configurations of the high temperature, disordered form I. Such a high energy barrier to the high temperature disordered form is unexpected. The temperature required to obtain the disordered polymorph is not far above room temperature, around 333 K to 343 K, and the crystal structure is very similar to the room temperature one. Our hypothesis is that this high energy barrier arises due to the small cell size (four molecules) in which we ran the MCT trajectories. A possibility is that such cell sizes cannot account for a lower energy transformation pathway between configurations that involves longer-range changes in structure. It could also be the case that the ordered models of polymorph I that we used, I_2_ and I_3_, are not representative of the true disordered crystal structure.

Shifting our focus to the disconnectivity graphs coloured by the packing motifs of the crystal structures (Fig. 6D–F), we can see that a large number of different packing motifs are accessible at low energy lids; this is more visible in the isoPAHAP (Fig. 6F) and PAHAP (Fig. 6E) graphs than in FIT (Fig. 6D), due to the lower number of local minima found in the MCT trajectories. This observation of low energy pathways between different packing motifs shows that the molecules in the low energy crystal structures are able to easily access different relative positions, which could relate to the appearance of a disordered polymorph in the system.

#### 3.3.4 Disconnectivity graphs of the whole sampled energy range

**Fig. 7:**
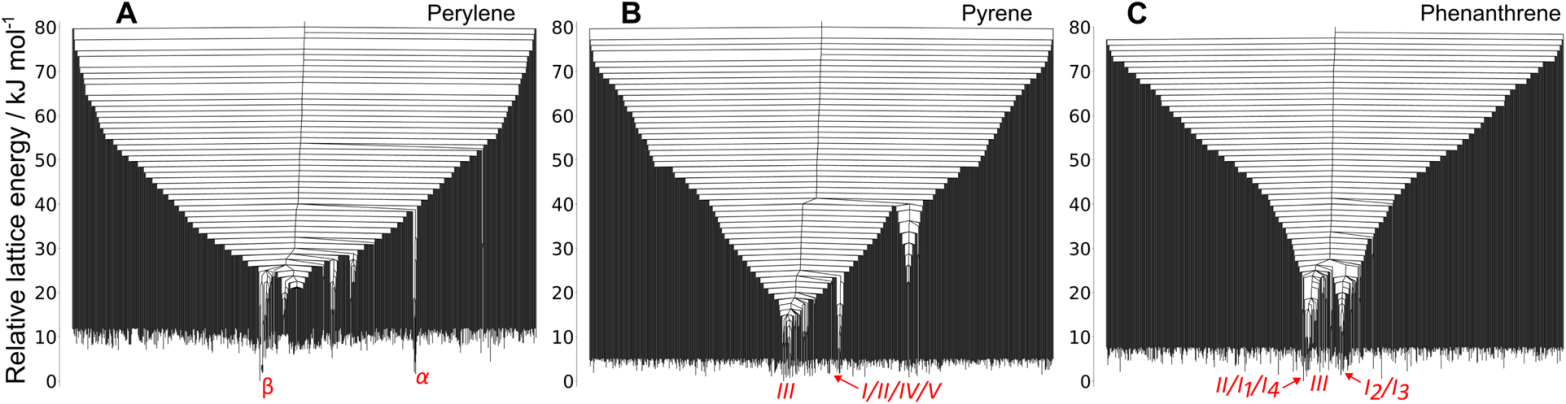
Disconnectivity graph of the top 700 lowest energy crystal structures from the MC trajectories of perylene (A), pyrene (B) and phenanthrene (C) with the PAHAP potential. In red we show the approximate location of the experimental matches. Many low energy structures can be found at high-energy lids. The same trend is seen in the other potentials, shown in SI S4.

The disconnectivity results shown up to this point have been limited to the starting crystal structures or just the low energy region of the landscape. However, our sampling spanned a larger energy range. In Fig. 7 we show the disconnectivity graphs for the 700 lowest energy structures found from the full sampling that was performed for each system, showing the results with the PAHAP potential. Similar graphs for the other potentials can be seen in SI Fig. S4.

We observe a large number of low energy structures that are separated by high energy barriers from the starting crystal structures: energy barriers higher than 70 kJ mol^−1^. Low energy structures with such high energy barriers are characteristic of frustrated systems,^43,44^ where the PES does not funnel towards a single low energy minimum, but where many low energy regions exist. Frustrated landscapes are typical of glassy systems.

The frustration of the landscapes is very clear for pyrene (Fig. 7B) and phenanthrene (Fig. 7C). In the perylene landscape (Fig. 7A) the effect is less pronounced, and the two lowest energy basins competing to be the global energy minimum correspond to the basins with the experimental structures. Although the global picture provided by the MCT simulations is of glassy landscapes, the regions containing observed crystal structures are slightly deeper than the rest of the low energy structures.

## 4 Computational cost

Having shown how the MCT algorithm can be employed to obtain further insights into the crystal PES of a molecular compound, we briefly discuss the cost of running these calculations. In Table 4 we provide a breakdown of the CPU hours spent on different parts of the workflow for the PAHAP potential; a breakdown of all three potentials is shown in SI Table S1.

**Table 4:** CPU hours on the University of Southampton Iridis 6 compute cluster (AMD Genoa EPYC 9654 CPUs) spent in CSP structure generation and clustering, and MCT trajectories, clustering and packing analysis with the PAHAP potential energy model. Data for the FIT and isoPAHAP potentials is provided in SI Table S1

	Perylene	Pyrene	Phenanthrene
Structure generation and minimization	1086	1628	858
Clustering	1	8	1
CSP total time	1087	1636	859
MC trajectories and minimization	42 021	43 083	40 082
Clustering	0	1	1
Packing analysis	12	44	14
MCT total time	42 033	43 128	40 097
MCT time per crystal structure	1314	1232	1114
Total time	43 120	44 764	40 956

Running the CSP calculations costs a total of 1087, 1636, and 859 CPU hours for perylene, pyrene and phenanthrene, respectively. The higher cost for pyrene is due to the extra search in SG *P*1̄ with Z′ = 2. The cost of running the MCT calculations amounts to 42 033, 43 128, and 40 097 CPU hours respectively. The MCT algorithm workflow is much more expensive computationally than CSP; apart from for pyrene, the cost of running the MCT sampling from a single starting crystal structure is greater than running the full CSP search for a single molecule.

The increased cost for running the MCT calculations is due to the nature of the algorithm. MCT involves first generating a trajectory by applying MC steps to a starting crystal structure, meaning that we have no room for parallelization in order to speed up the job, as each step in the trajectory depends on the previous. The majority of the time spent on each MCT step is spent on the energy evaluation of the perturbed structure, to determine whether the configuration is above the set energy threshold. This bottleneck involving the energy evaluations can be addressed. One approach is to decrease the cost of energy evaluation by applying lower cost energy models. Energy evaluations with the anisotropic atom–atom functional form of the three potentials applied in this work is approximately 10× the computational cost of isotropic atom–atom force fields. Software changes could also decrease the computational expense: in the current, developmental applications of MCT, the DMACRYS^30^ software is called individually for each energy evaluation, which involves a relatively large overhead of initialising each energy evaluation. Improved software integration will lower these costs.

## 5 Beyond PAHs

This study has focused on small rigid molecules with extensive experimental data in the literature, but the use of the MCT algorithm can be extended to other types of molecular crystals. Here, we briefly mention some cases in which the use of the MCT algorithm can be beneficial, and compare the challenges to what we have presented with PAHs.

Many applications will require extending the Monte Carlo sampling method to flexible molecules. For this, internal molecular degrees of freedom, such as bond angles and dihedrals, must be defined in the MC move set and an energy model capable of handling intramolecular energy changes must be used. The increased number of degrees of freedom for a flexible-molecule system compared to rigid-molecule simulations will require greater sampling per lid to ensure thorough sampling of the PES. As shown in Section 4, the cost of the MCT trajectories is already significant for rigid-molecule crystal structures. Furthermore, given the general failure of off-the-shelf force fields for CSP of flexible molecules, an accurate description of the PES of flexible-molecule crystal structures will require higher cost energy models. Dispersion-corrected solid state DFT is often successful for CSP,^6^ but is too costly for applications within MCT. However, machine learning methods show promise for molecular CSP^4,45^ and should be evaluated for applications in MCT simulations. The increased sampling and higher cost of energy evaluations will initially necessitate ways of lowering computational cost, such as limiting simulations to a lower energy lid range or larger lid increments than what is possible for rigid-molecule systems.

The MCT method can be applied to molecular systems for which we have little or no experimental data. The only experimental information used in the PAH studies presented here was to ensure that CSP matches to observed polymorphs were included in the set of starting points for MCT trajectories. The MCT algorithm provides information on the depth of the local energy minimum of different crystalline arrangements, which can be used to assess whether a putative crystal structure can be obtained through experiment on the basis that deep lying minima on the landscape can result in stable crystals. Applying the MCT algorithm as we have done with PAHs (the 3-stage sampling defined in Section 2.2) could provide a global picture of the crystal PES, which could identify interesting regions of the landscape (*e.g.* the basins containing the experimental polymorphs of the perylene molecule in Fig. 7A).

## 6 Conclusion

By combining crystal structure prediction with Monte Carlo threshold simulations, we have carried out an analysis of the crystal packing potential energy surface of three polymorphic PAH molecules: phenanthrene, pyrene and perylene. The traditional CSP workflow allows us to find the relative energy of the experimental polymorphs, but CSP results lack the information to be able to understand the appearance of the polymorphs under different experimental conditions. The Monte Carlo threshold algorithm provides us with the energy barriers between the local minima in the PES, enabling analysis of the shape and connectivity of the energy basins. In this study, we have shown how this information helps rationalize the experimental conditions required to transform between the known polymorphs of each molecule.

For perylene, we find that the energy basins containing the experimental polymorphs have large energy barriers separating them. Furthermore, the basins have structures with lower energy than the rest of the energy landscape, which explains why both polymorphic forms are observed under the same conditions and why high temperature is needed to convert between them. In the case of pyrene, we observe two basins in the landscape, one containing polymorph III and another basin containing the rest of the known polymorphs; the structure of the energy landscape revealed by the simulations explains why form III has to be accessed either using high temperature to overcome this energy barrier, or using recrystallisation to avoid the energy barrier, while transitions between the other polymorphs can occur in the solid state at ambient temperature. Finally, in the phenanthrene landscape there is a large number of structures with different packing motifs at low energy lids, which we can relate to the fact that the molecules in the crystal can easily shift and rotate and could explain the appearance of a disordered polymorph. Accounting for all the local minima found in our MCT trajectories, we observe that the crystal energy landscapes show a large degree of frustration: many low energy structures have high energy barriers separating them. This is a behaviour expected of glassy systems, yet the three molecules we have studied readily crystallise.

The weak dependence of these findings on the model potential adds to our confidence in the conclusions drawn from CSP and MCT simulations. The disconnectivity graphs for each molecule show that the connections between polymorphs and the energy barriers separating them are very similar between the three potential energy models. But, accounting for all local minima found, both in the CSP and MCT searches, there are noticeable differences. PAHAP and isoPAHAP, potentials specific to PAHs, show a lower number of local minima found than the amount observed with the FIT potential. This means that the PESs defined by PAHAP and isoPAHAP are smoother than the one defined by FIT; this might be related to FIT's parametrization as a transferrable potential that can be used with many different chemical systems. Although the MCT algorithm can deal with PESs of any ruggedness, potential energy models designed for specific molecular compounds should be preferred over general transferrable potentials, where available.

This study demonstrates the value of mapping energy barriers between predicted crystal structures resulting from CSP. Thus, we believe that the approach presented in this work is valuable as a complementary method to CSP studies of polymorphism and, while there are challenges involved with generalising the method to more complex molecular crystals, the approach has great potential value in solid form screening (*e.g.* for pharmaceutical materials) and for guiding functional materials discovery.

## Author contributions

Pedro Juan-Royo: software, data curation, investigation, formal analysis, validation, writing – original draft preparation, writing – reviewing and editing, visualization Graeme M. Day: conceptualization, supervision, writing – reviewing and editing.

## Conflicts of interest

There are no conflicts to declare.

## Supplementary Material

SC-OLF-D5SC08644B-s001

## Data Availability

The CSP landscape and disconnectivity graph data produced in this work is provided under a CC-BY license at https://doi.org/10.5258/SOTON/D3710. Supplementary information (SI): a detailed description of the methodology^21–28,30,31,36,38,40,46–54^ is described in the SI along with SI figures showing the full CSP landscapes and more visualisations of the disconnectivity graphs. See DOI: https://doi.org/10.1039/d5sc08644b.
