# Supplementary material for: From crystal structure prediction to polymorphic behaviour: Monte Carlo threshold mapping of crystal energy landscapes
Source: Chem Sci. 2026 Jan 12;17(7):3480–91. doi: 10.1039/d5sc08644b (PMC12834216; doi:10.1039/d5sc08644b)
Supplement: SC-017-D5SC08644B-s001 [file SC-017-D5SC08644B-s001.pdf]

Supplementary Information (SI) for Chemical Science.
This journal is © The Royal Society of Chemistry 2026

Supplementary Information for "From Crystal Structure Prediction to Polymorphic Behaviour: Monte Carlo Threshold Mapping of Crystal Energy Landscapes"

Pedro Juan-Royo[a] Graeme M. Day*[,a]

Contents

S1 Computational methods **1**
S1.1 Crystal structure prediction 1
S1.2 Monte Carlo threshold algorithm 2
S1.3 Potential energy models 5
S1.4 Experimental structures 5
S1.5 Crystal packing analysis 8
S2 Energy-density landscapes **11**
S3 Disconnectivity graphs **13**

S1 Computational methods

S1.1 Crystal structure prediction

Firstly, molecular geometries were optimized at the B3LYP / 6-311** level of theory in Gaussian09[1]. These optimized geometries were kept fixed throughout the calculations. Distributed atom-centered multipoles up to hexadecapole were generated from the optimized electronic density using GDMA[2], and partial charges were fitted to the multipoles using the MULFIT[3] program.

The structure generation stage was carried out using our GLEE[4] program, as implemented in mol-CSPy[5]. Crystal structures were generated in the top 10 most common molecular organic space groups (SGs) with Z′ = 1: $Pbca$, $P2_1/c$, $C2/c$, $P2_12_12_1$, $P\bar{1}$, $P2_1$, $Pna2_1$, Cc, $Pca2_1$ and $C2$. The structure generation search terminated once 10 000 structures were generated and successfully energy minimized for each SG. For the pyrene molecule, an extra crystal generation stage was performed with Z′ = 2 in SG $P\bar{1}$ targeting 40 000 structures. This was done to find matching structures to the experimental polymorph IV[6].

The generated structures were lattice-energy minimized in a 3-stage procedure, using the PMIN[7] and DMACRYS[8] software packages, consisting of: an initial PMIN minimization at zero pressure and partial charge electrostatics, following with a DMACRYS minimization with distributed (atomic) multipole electrostatics and at 0.1 GPa, and finally a DMACRYS minimization at zero pressure with multipole electrostatics.

The potential energy models used for the minimizations were: FIT[9], PAHAP[10] and isoPAHAP[11]. These are described in Section S1.3.

After the target structures were generated, the set of structures was clustered to remove any duplicates. First, we compared simulated powder X-ray diffraction (pXRD) patterns generated with

a *School of Chemistry and Chemical Engineering*, University of Southampton, Southampton, SO17 1BJ, UK
* g.m.day@soton.ac.uk

the PLATON[12] package; comparisons were performed with a constrained dynamic time warping algorith. This was followed with the COMPACK[13] algorithm as it is implemented in the Cambridge structural database (CSD)[14] API, using a cluster size of 30 molecules, angle and distance tolerance of 30° and 30 % respectively, and a threshold on root mean squared deviations in atomic positions of 0.3 Å. For efficiency, only structures within 1.0 kJ mol^{-1} in lattice energy and 0.05 g cm^3 in density were compared at both steps.

Table S1 shows the cost of running the crystal structure prediction (CSP) workflow and the subsequent clustering of duplicate structures. The data for pyrene includes the extra structures generated with $Z' = 2$. Structure generation with the PAHAP potential takes the longest due to the nature of the potential energy model (anisotropic atom-atom repulsion-dispersion). Clustering of the pyrene structures takes longer than in the other systems due to the extra structures generated in the $Z' = 2$ search.

S1.2 Monte Carlo threshold algorithm

The Monte Carlo threshold (MCT) trajectories were started from the crystal structures of the CSP landscape that matched the experimental polymorphs of each system, plus an extra 30 of the lowest energy predicted crystal structures. These 30 additional structures were selected using a generalised convex hull (GCH) algorithm martin2025adapted. The structures of the CSP landscape were ranked by their distance to the GCH, and 30 structures were selected starting from the ones closest to the GCH, skipping those that were above 15 kJ mol^{-1} from the global minimum of the CSP landscape. If one of the structures to be selected with the GCH ranking was also a match to an experimental polymorph, it was skipped and the next closest to the GCH was selected instead.

Once the structures were selected, they were expanded to $P1$ Niggli-reduced cells in order to remove any symmetry constraints on the sampling and avoid running the algorithm in stretched cells that can arise from the CSP minimization steps, as well as using the smallest possible unit cell representation of each crystal structure. Whenever possible, the MCT trajectories were run in cells with 4 molecules. Any supercells that had to be generated were done by doubling the original cell along the shortest cell axis. A few structures had smallest cells with 8 molecules, in which case twice as much sampling was performed than in the cells with 4 molecules.

A total of three independent MCT trajectories were run from each structure, each one with an increasing amount of sampling of the lower energy regions of the potential energy surface (PES). The three sampling schemes, S1, S2, and S3, are shown in Table S2. S1 and S2, using 5.0 and 2.5 kJ mol^{-1} lid increments, are used to sample up to high energies above the starting point to ensure connections between all crystal structures are found. S3, using small lid increments of 1.25 kJ mol^{-1} and more extensive sampling, is used to get a very thorough sampling of the energy regions near the starting points and find the lowest-energy connections possible.

The single-point energy calculation after every Monte Carlo (MC) step was done in DMACRYS with multipole electrostatics. The available MC moves are shown in Table S3. At each step, only one of the available move types is randomly selected and performed. The probabilities for each move type were calculated as the proportion of the total degrees of freedom of the system.

After each accepted MC step, the perturbed structure was energy-minimized following a 3-stage procedure similar to the one done with the CSP structures: an initial DMACRYS minimization at ambient pressure and multipole electrostatics, following with a DMACRYS minimization with distributed multipole electrostatics and at 0.1 GPa, and finally a DMACRYS minimization at ambient pressure with multipole electrostatics. In this case, we replace the PMIN with partial charges minimization with a DMACRYS minimization with multipole electrostatics, to avoid possible cases where the change in the energy model samples a different PES and could therefore ”jump” to otherwise inaccessible basins.

Table S1 shows the cost of running the MCT algorithm on the selected structures in each system. The cost is significantly higher than running CSP on the same systems: running the MCT algorithm on a single crystal structure takes more CPU hours than generating the whole CSP landscape.

Table S1: CPU hours in the Iridis 6 compute cluster (AMD Genoa EPYC 9654 CPUs) spent in CSP structure generation and clustering, and MCT trajectories, clustering and packing analysis.

	Perylene			Pyrene			Phenanthrene		
	FIT	PAHAP	isoPAHAP	FIT	PAHAP	isoPAHAP	FIT	PAHAP	isoPAHAP
Structure generation and minimization	697	1086	634	695	1628	1095	580	858	540
Clustering	1	1	1	17	8	13	1	1	1
CSP total time	698	1087	635	712	1636	1108	581	859	541
MC trajectories and minimization	39 044	42 021	27 943	46 274	43 083	34 919	38 361	40 082	38 305
Clustering	0	0	0	2	1	1	1	1	1
Packing analysis	3	12	9	109	44	18	6	14	6
MCT total time	39 047	42 033	27 952	46 385	43 128	34 938	38 368	40 097	38 312
MCT time per crystal	1220	1314	874	1325	1232	998	1066	1114	1064
Total time	39 745	43 120	28 587	47 097	44 764	36 046	38 949	40 956	38 853

Table S2: Description of the three sampling schemes used for the MC trajectories of the treshold algorithm. S1 and S2 reach the same energy-lid above the starting structure, but S2 has twice the number of MC steps. S3 does not reach as high an energy-lid above the starting point, but it is used to thoroughly explore the low energy region of the PES to find the lowest energy connections possible.

Sampling scheme	Total MC moves	MC moves per lid	Lid energy increases / $\mathrm{kJ\,mol^{-1}}$	Max. lid energy / $\mathrm{kJ\,mol^{-1}}$
S1	13 000	1000	5.00	65.00
S2	26 000	1000	2.50	65.00
S3	34 000	2000	1.25	21.25

Table S3: The moveset for the MC perturbations. The probability of performing one of the moves was calculated as the proportion to the total degrees of freedom of the system. The changes in cell volume depend on the number of molecules in the cell.

Move	Maximum move amount
Translation	0.5 Å
Rotation	0.05°
Cell lengths	0.5 Å
Cell angles	0.5 Å
Cell volume (per molecule)	25 Å^3

Connections between MCT trajectories were found by clustering the structures using simulated pXRD patterns generated by PLATON. The COMPACK algorithm was not used in this case due to the large number of structures generated in the trajectories. From the connections found between trajectories, disconnectivity graphs could be constructed.

It was observed that once the selected structures were minimized in the $P1$ cells, some of them coalesced to the same minima and were clustered together when applying the pXRD comparison. This means that the number of initial structures in the disconnectivity graphs might not add up to 30 plus the experimental matches. The total number of minima for each system and potential is shown in Table S4, as well as the maximum number of structures possible if no minima coalesced in the $P1$ PES.

Table S4: Number of unique MCT trajectory starts after pXRD clustering of the $P1$ energy-minimized cells for every potential energy model used. The last row shows the maximum number of trajectory starts that can be per system. Pyrene experimental polymorphs I and II are clustered together when energy-minimized with the FIT and PAHAP potentials in the $P1$ SG. Phenanthrene polymorphs II, I_1 and I_4 also cluster together, and I_2 and I_3 as well.

Potential	Phenanthrene	Pyrene	Perylene
FIT	33	34	30
PAHAP	32	34	30
isoPAHAP	31	35	28
Maximum	34	34/34/35	32

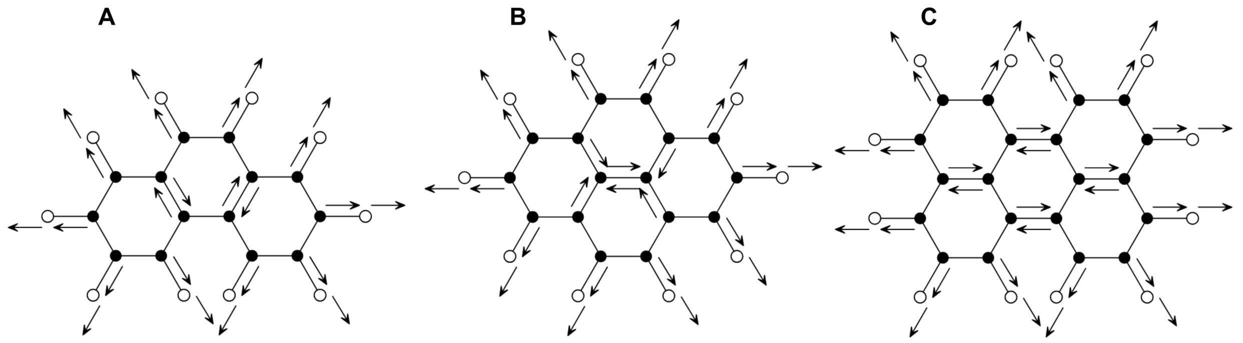

Figure S1: Directions of the z-axis for each atomic site of the three molecules in this study, required for the anisotropic atom-atom repulsion-dispersion potential. The atomic axes for pyrene are taken from[10].

S1.3 Potential energy models

Three different potential energy models were employed when energy-minimizing the CSP and MCT generated structures and doing single-point energy calculations of the MC perturbations: FIT[15], PAHAP[10] and isoPAHAP[11], each combined with atomic multipole electrostatics. FIT is a transferable potential energy model for organic molecular crystal structure modelling, and is therefore parametrized from a variety of molecular chemistries. PAHAP is an anisotropic atom-atom potential parametrized exclusively with data of polycyclic aromatic hydrocarbons (PAHs) dimers, and isoPAHAP is an isotropic potential derived from it.

FIT and isoPAHAP are isotropic atom-atom potentials of the Buckingham form shown in Equation (1).

$$V_{iso}(r) = A\exp(-Br) - \frac{C}{r^6} \tag{1}$$

Where A, B and C are constants whose values depend on the species that interact. The PAHAP potential is an anisotropic atom-atom potential of the Buckingham form with a damped dispersion term $f(r)$, as shown in Equation (2).

$$V_{aniso}(r) = A\exp\Big[-B\big(r-\rho(\Omega)\big)\Big] - f(r)\frac{C}{r^6} \tag{2}$$

Compared to the isotropic potential, $\rho(\Omega)$ is a function that depends on the relative orientation of the two interacting atomic sites with respect to defined atomic axis. These are shown in Figure S1.

The use of atom-centered multipole electrostatics means that even if the non-electrostatic part of the potentials is isotropic (FIT and isoPAHAP), when including the electrostatic term to the energy calculations, all three potentials have an anisotropic atom-atom form.

S1.4 Experimental structures

The crystal files for experimental polymorphs were taken from different sources. Perylene polymorphs were taken from the CSD, refcode PERLEN01[16] for the α polymorph and PERLEN07[17] for the β one. The CSD entry for polymorph β has $Z' = 0.5$ in SG $P2_1/c$, which can't be handled with our workflow, so the SG symmetry was changed to subgroup $P2_1$ with $Z' = 1$.

Pyrene polymorphs I, II, IV and V were all taken from the data provided in the high pressure study of pyrene by Zhou *et al.*[6], while the file for polymorph III was taken from the CSD entry with refcode PYRENE08[18]. The CSD entry for polymorph III has $Z' = 0.5$. Due to the limitations in our workflow to handle non-integer number of formula units in the asymmetric unit, we changed the SG symmetry of the crystal from $P2_1/a$ to $P2_1$ with one whole molecule in the asymmetric unit.

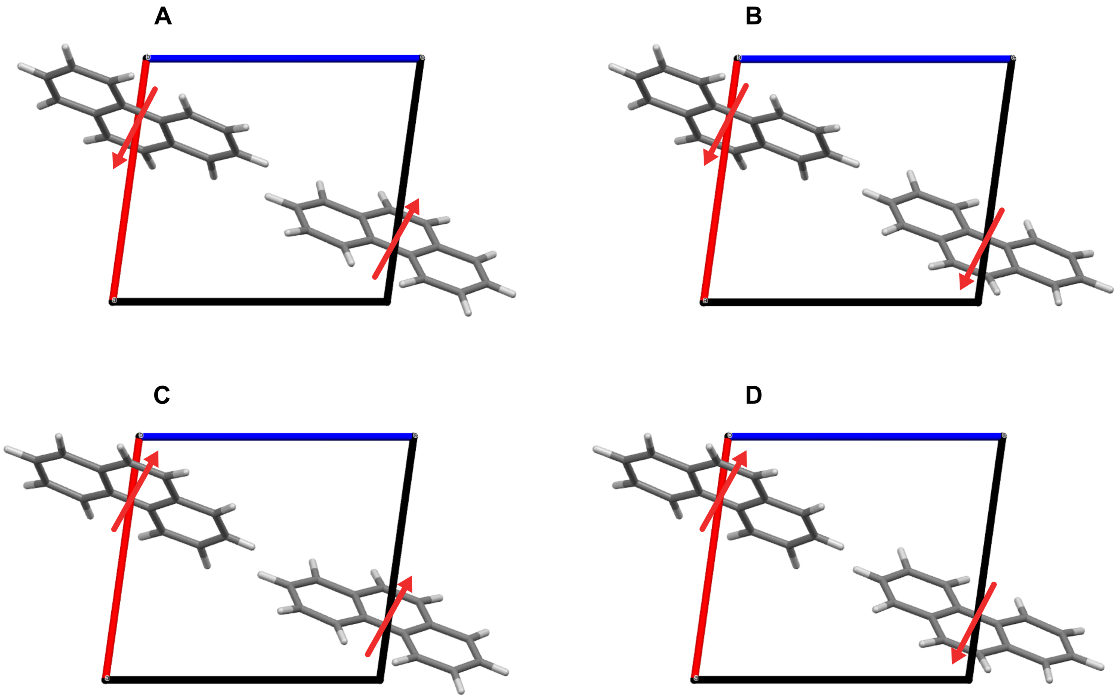

Figure S2: The four crystals generated for the disordered polymorph I of phenanthrene. Red arrows show the orientation of the molecules in the crystals. A is I_1, B I_2, C I_3, and D I_4. A and D are equivalent due to symmetry, B and C are equivalent too.

Phenanthrene polymorphs II and III were taken from CSD entries PHENAN04[19] and PHENAN14[18] respectively. No file with resolved atomic positions for polymorph I was found in the CSD. This polymorph packs similarly to polymorph I but has orientational disorder: molecules are oriented in two possible directions related by an inversion center in the molecule[20]. To create a crystal file for this polymorph we used the unit cell data from the PHENAN11[20] CSD entry. Combinations of the possible orientations of the two molecules in the unit cell were created, resulting in 4 crystal files shown in Figure S2: I_1, I_2, I_3, I_4. Due to symmetry, I_1 is equivalent to I_4, and I_2 equivalent to I_3. Due to the symmetry of the I_2 and I_3 crystals and the SGs and Z' values searched during CSP, no matches could be found in the CSP landscapes.

In order to search the CSP landscape for structures matching the experimental polymorphs, we replaced the molecules in the experimental crystals with the B3LYP / 6-311** geometry-optimized ones used in the CSP crystal generation step. The crystals were then energy-minimized with the three potential energy models. The original unit cell parameters and the energy-minimized ones are shown in Table S5.

Table S5: Unit cell parameters of original polymorphs and energy-minimized versions of the experimental polymorphs with each potential.

Polymorph	a/Å	b/Å	c/Å	$\alpha/°$	$\beta/°$	$\gamma/°$
Perylene α[16]	11.28	10.83	10.26	90.00	100.55	90.00
Perylene α FIT	10.20	11.21	11.00	90.00	97.59	90.00
Perylene α PAHAP	10.11	10.55	10.91	90.00	96.72	90.00
Perylene α isoPAHAP	9.96	10.96	10.92	90.00	96.62	90.00
Perylene β[17]	9.75	5.82	10.58	90.00	96.69	90.00
Perylene β FIT	10.06	5.46	11.38	90.00	100.07	90.00
Perylene β PAHAP	9.96	5.26	11.05	90.00	99.48	90.00

Continued on next page

Table S5: Unit cell parameters of original polymorph and energy-minimized ones. (*cont.*)

Polymorph	a/Å	b/Å	c/Å	α/°	β/°	γ/°
Perylene β isoPAHAP	9.80	5.57	10.88	90.00	98.34	90.00
Pyrene I[6]	8.48	9.26	13.66	90.00	100.31	90.00
Pyrene I FIT	8.06	10.04	13.06	90.00	93.01	90.00
Pyrene I PAHAP	7.98	9.90	12.31	90.00	92.07	90.00
Pyrene I isoPAHAP	8.00	10.16	12.67	90.00	101.69	90.00
Pyrene II[6]	8.14	9.86	12.11	90.00	96.48	90.00
Pyrene II FIT	8.06	10.04	13.06	90.00	93.02	90.00
Pyrene II PAHAP	7.98	9.90	12.31	90.00	92.06	90.00
Pyrene II isoPAHAP	7.92	9.95	12.64	90.00	93.43	90.00
Pyrene III[21]	15.35	3.85	8.65	90.00	103.30	90.00
Pyrene III FIT	15.85	3.88	8.49	90.00	102.63	90.00
Pyrene III PAHAP	15.24	3.78	8.36	90.00	101.94	90.00
Pyrene III isoPAHAP	13.02	4.85	7.89	90.00	97.73	90.00
Pyrene IV[6]	7.59	10.22	11.19	92.54	95.04	91.21
Pyrene IV FIT	7.88	10.45	12.92	92.43	93.20	91.07
Pyrene IV PAHAP	7.71	10.30	12.30	92.58	92.16	90.61
Pyrene IV isoPAHAP	7.68	10.35	12.63	92.54	92.58	91.05
Pyrene V[6]	7.45	6.45	16.10	90.00	100.65	90.00
Pyrene V FIT	8.01	7.75	17.19	90.00	99.81	90.00
Pyrene V PAHAP	7.90	7.46	16.65	90.00	99.11	90.00
Pyrene V isoPAHAP	7.70	7.75	16.92	90.00	98.92	90.00
Phenanthrene I[20]	8.51	6.22	9.53	90.00	98.73	90.00
Phenanthrene I_1 FIT	8.66	6.02	9.39	90.00	95.10	90.00
Phenanthrene I_1 PAHAP	8.68	5.61	9.30	90.00	95.75	90.00
Phenanthrene I_1 isoPAHAP	8.58	5.79	9.25	90.00	95.30	90.00
Phenanthrene I_2 FIT	8.86	5.87	9.62	89.95	94.21	89.99
Phenanthrene I_2 PAHAP	8.83	5.52	9.49	89.94	94.53	89.99
Phenanthrene I_2 isoPAHAP	8.69	5.72	9.44	89.95	94.08	89.99
Phenanthrene I_3 FIT	8.86	5.87	9.62	90.05	94.21	90.01
Phenanthrene I_3 PAHAP	8.83	5.52	9.49	90.06	94.53	90.01
Phenanthrene I_3 isoPAHAP	8.69	5.72	9.44	90.05	94.08	90.01
Phenanthrene I_4 FIT	8.66	6.02	9.39	90.00	95.10	90.00
Phenanthrene I_4 PAHAP	8.68	5.61	9.30	90.00	95.75	90.00
Phenanthrene I_4 isoPAHAP	8.58	5.79	9.25	90.00	95.30	90.00
Phenanthrene II[19]	8.46	6.16	9.47	90.00	97.70	90.00
Phenanthrene II FIT	8.66	6.02	9.39	90.00	95.11	90.00
Phenanthrene II PAHAP	8.68	5.61	9.30	90.00	95.75	90.00
Phenanthrene II isoPAHAP	8.58	5.79	9.25	90.00	95.30	90.00
Phenanthrene III[18]	12.94	3.82	17.69	90.00	99.13	90.00
Phenanthrene III FIT	13.30	3.94	18.15	90.00	99.03	90.00
Phenanthrene III PAHAP	12.88	3.83	17.82	90.00	97.96	90.00
Phenanthrene III isoPAHAP	9.51	5.60	17.09	90.00	88.11	90.00

Matches in the CSP landscape were found using the CSD energy-minimized crystal structures with the COMPACK algorithm as implemented in the CSD API, using a cluster size of 30 molecules, a cutoff in RMSD of atomic positions of 0.30 Å, and angle and distance tolerance of

30.00° and 30.00 % respectively. The COMPACK algorithm provides the root-mean-square deviation ($RMSD_{30}$) as a measure of how closely the structures match. The matching structures, their SG and the $RMSD_{30}$ between them and the experimental ones is shown in Table S6.

It must be noted that pyrene polymorphs I and II are clustered together when energy-minimized using the FIT and PAHAP potentials. Phenanthrene polymorphs II, I_1 and I_4 cluster together, as well as I_2 and I_3.

S1.5 Crystal packing analysis

Analysis of the packing of the crystals from the disconnectivity graphs was done using the Autopack[22] python package. The settings used were the default ones, shown in Table S7.

Table S6: SG and $RMSD_{30}$ (in Å) of the CSP landscape structures matching experimental polymorphs. Here, $RMSD_{30}$ compares the CSP structures to the energy-minimized versions of the experimental structures. The SG of the experimental polymorphs are provided for reference. Pyrene III and perylene β polymorphs have experimental crystals with $Z' = 0.5$, in brackets is the subgroup we used in our calculations. All $P\bar{1}$ matches in pyrene are found in the $Z' = 2$ CSP search.

			FIT matches		PAHAP matches		isoPAHAP matches	
Molecule	Polymorph	Experimental SG	SG	$RMSD_{30}$	SG	$RMSD_{30}$	SG	$RMSD_{30}$
Phenanthrene	I_1/I_4	$P2_1$	$P2_1$	2.3×10^{-3}	$P2_1$	1.6×10^{-3}	$P2_1$	1.2×10^{-3}
	I_2/I_3	$P2_1$						
	II	$P2_1$	$P2_1$	2.9×10^{-4}	$P2_1$	6.9×10^{-4}	$P2_1$	1.3×10^{-3}
	III	$P2_{1/n}$	$P2_1/c$	4.9×10^{-4}	$P2_1/c$	2.3×10^{-4}	$P2_1/c$	4.2×10^{-3}
Pyrene	I	$P2_1/c$	$P\bar{1}$	2.6×10^{-1}	$P\bar{1}$	2.4×10^{-3}	$P\bar{1}$	8.5×10^{-4}
	II	$P2_1/c$	$P\bar{1}$	2.6×10^{-1}	$P\bar{1}$	1.4×10^{-3}	$P\bar{1}$	1.3×10^{-3}
	III	$P2_1/a$ $(P2_1)$	$P2_1/c$	4.8×10^{-3}	$P2_1$	8.2×10^{-3}	$P\bar{1}$	3.5×10^{-3}
	IV	$P\bar{1}$	$P\bar{1}$	1.6×10^{-3}	$P\bar{1}$	6.3×10^{-4}	$P\bar{1}$	1.8×10^{-3}
	V	$P2_1/c$	$P2_1/c$	4.8×10^{-3}	$P\bar{1}$	2.8×10^{-3}	$P\bar{1}$	3.6×10^{-3}
Perylene	α	$P2_1/a$	$P2_1/c$	3.7×10^{-3}	$P2_1/c$	1.6×10^{-3}	$P2_1/c$	1.2×10^{-3}
	β	$P2_1/c$ $(P2_1)$	$P2_1$	3.2×10^{-4}	$P2_1$	5.8×10^{-4}	$P2_1$	1.7×10^{-3}

Table S7: Autopack[22] settings used for packing analysis of the disconnectivity graph structures.

Setting name	Value
threshold	120
restarts	4
z_penalty	0.0315
alpha	1.20
in_plane	25.00°

S2 Energy-density landscapes

The energy-density plots of the CSP landscapes can be seen in Figure S3. The experimental polymorph matches are shown with red squares, the rest of the generated structures are shown in black crosses.

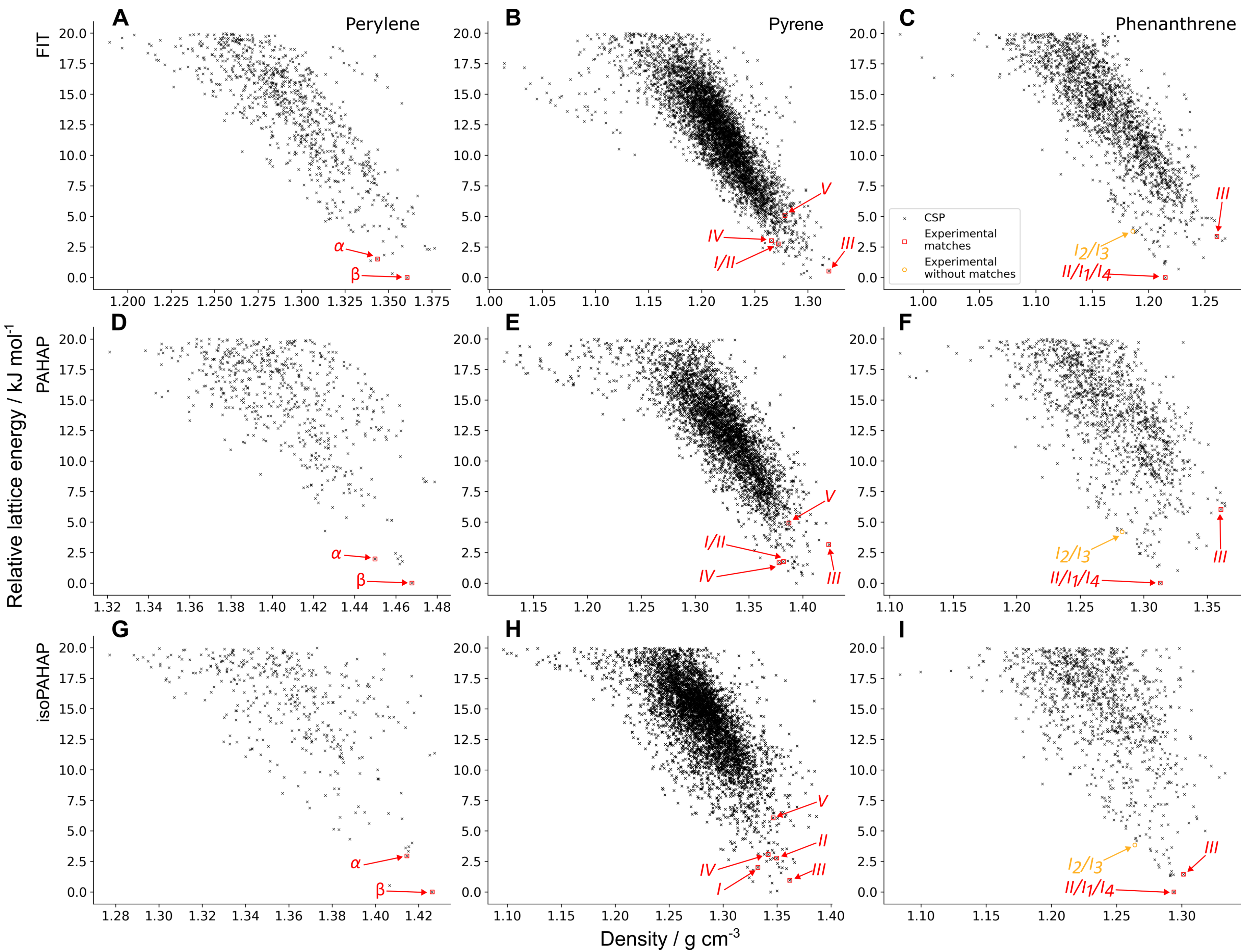

Figure S3: Energy-density plots of the CSP landscapes of the three molecules: perylene (A,D,G), pyrene (B,E,H) and phenanthrene (C,F,I). The landscapes have been truncated to show only the structures within $20.00\,\mathrm{kJ\,mol^{-1}}$ of the global energy minimum. Black crosses are all the structures generated with the CSP workflow. Red squares indicate the structures that match an experimental crystal, orange circles are the experimental crystal structures that had no matches in the landscape.

S3 Disconnectivity graphs

The top 700 lowest energy structures of each system and potential generated with the MCT algorithm are shown in Figure S4. We can observe that, at high-energy lids above the starting structures for the MCT trajectories, a large number of low energy structures are found.

The full disconnectivity graphs for all molecules and potential energy models are shown in Figure S5.

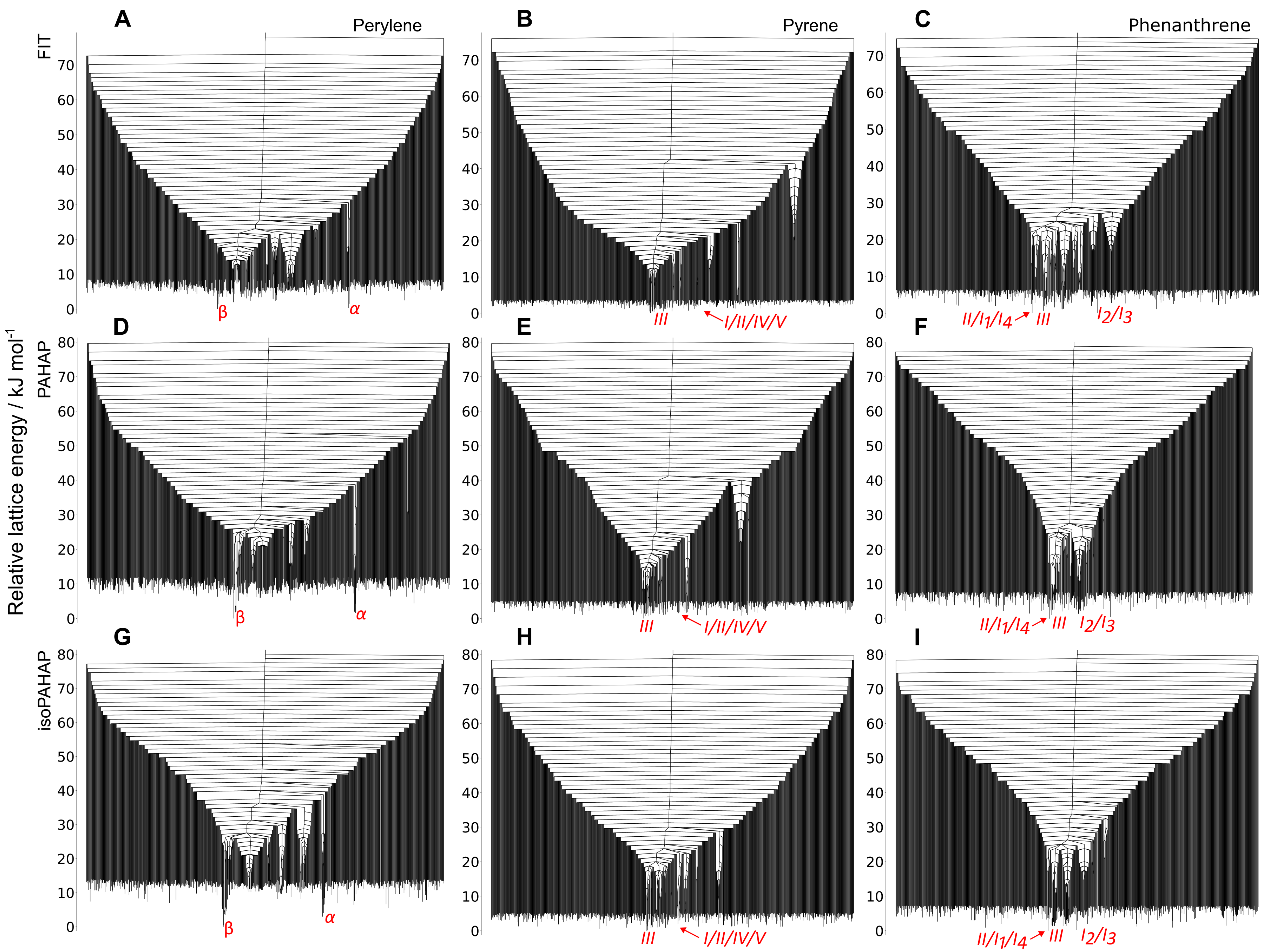

Figure S4: Disconnectivity graphs showing the top 700 lowest energy structures from all MCT trajectories for perylene (A,D,G), pyrene (B,E,H) and phenanthrene (C,F,I). In red we show the approximate location of the experimental matches. Many low energy structures can be found at high-energy lids indicating a high degree of frustration.

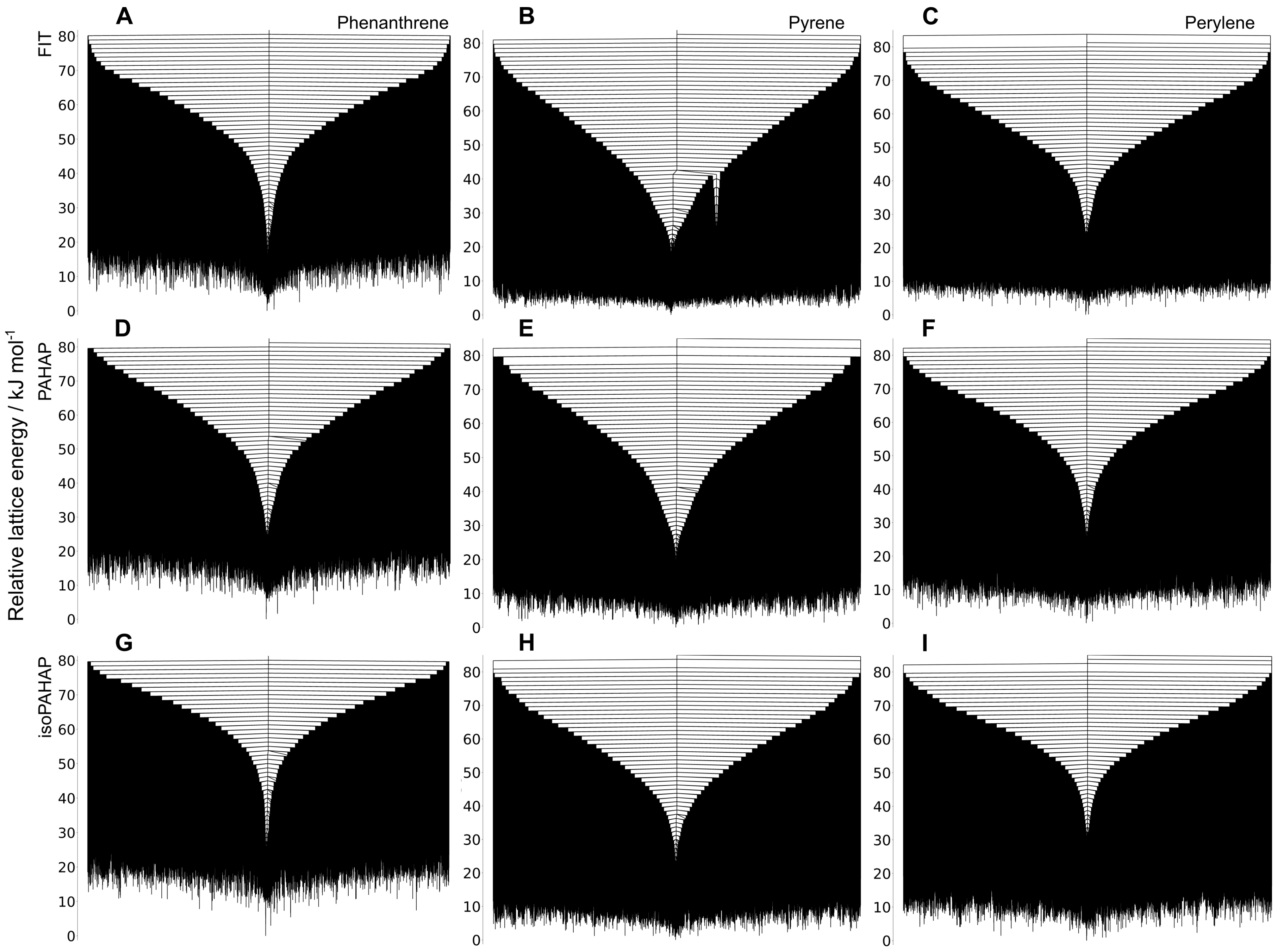

Figure S5: Full disconnectivity graphs for perylene (A,D,G), pyrene (B,E,H) and phenanthrene (C,F,I).

References

[1] M. J. Frisch *et al.*, *Gaussion09*, Revision D.01.

[2] A. J. Stone, *J. Chem. Theory Comput.*, 2005, **1**, 1128–1132.

[3] G. G. Ferenczy, *J. Comput. Chem.*, 1991, **12**, 913–917.

[4] D. H. Case, J. E. Campbell, P. J. Bygrave and G. M. Day, *J. Chem. Theory Comput.*, 2016, **12**, 910–924.

[5] G. M. Day, J. Bramley, P. W. V. Butler, P. J. Bygrave, D. H. Case, C. Y. Cheng, R. Cuadrado, J. Dickman, J. Dorrell, J. Glover, R. Hafizi, J. Johal, D. P. McMahon, J. Nyman, P. R. Spackman, C. R. Taylor, J. Yang and S. Yang, *mol-CSPy*, `https://gitlab.com/mol-cspy/mol-cspy`, development version.

[6] W. Zhou, Y. Yin, D. Laniel, A. Aslandukov, E. Bykova, A. Pakhomova, M. Hanfland, T. Poreba, M. Mezouar, L. Dubrovinsky and N. Dubrovinskaia, *Commun. Chem.*, 2024, **7**, 209.

[7] J. R. Holden, Z. Du and H. L. Ammon, *J. Comput. Chem.*, 1993, **14**, 422–437.

[8] S. L. Price, M. Leslie, G. W. Welch, M. Habgood, L. S. Price, P. G. Karamertzanis and G. M. Day, *Phys. Chem. Chem. Phys.*, 2010, **12**, 8478–8490.

[9] D. t. Williams and S. Cox, *Acta Crystallogr., Sect. B:Struct. Sci., Cryst. Eng. Mater.*, 1984, **40**, 404–417.

[10] T. S. Totton, A. J. Misquitta and M. Kraft, *J. Chem. Theory Comput.*, 2010, **6**, 683–695.

[11] T. S. Totton, A. J. Misquitta and M. Kraft, *Phys. Chem. Chem. Phys.*, 2012, **14**, 4081–4094.

[12] A. Spek, *Appl. Crystallogr.*, 2003, **36**, 7–13.

[13] J. A. Chisholm and S. Motherwell, *Appl. Crystallogr.*, 2005, **38**, 228–231.

[14] C. R. Groom, I. J. Bruno, M. P. Lightfoot and S. C. Ward, *Acta Crystallogr., Sect. B:Struct. Sci., Cryst. Eng. Mater.*, 2016, **72**, 171–179.

[15] D. S. Coombes, S. L. Price, D. J. Willock and M. Leslie, *J. Phys. Chem.*, 1996, **100**, 7352–7360.

[16] A. Camerman and J. Trotter, *Proc. R. Soc. London, Ser. A*, 1964, **279**, 129–146.

[17] A. Ranganathan and G. Kulkarni, *J. Chem. Sci. (Berlin, Ger.)*, 2003, **115**, 637–647.

[18] F. P. A. Fabbiani, D. R. Allan, W. I. F. David, S. A. Moggach, S. Parsons and C. R. Pulham, *CrystEngComm*, 2004, **6**, 505–511.

[19] D. Jones and J. Yerkess, *J. Cryst. Mol. Struct.*, 1971, **1**, 17–23.

[20] V. Petříček, I. Císařová, L. Hummel, J. Kroupa and B. Březina, *Acta Crystallogr., Sect. B:Struct. Sci.*, 1990, **46**, 830–832.

[21] F. P. A. Fabbiani, D. R. Allan, S. Parsons and C. R. Pulham, *Acta Crystallogr., Sect. B:Struct. Sci.*, 2006, **62**, 826–842.

[22] D. Loveland, B. Kailkhura, P. Karande, A. M. Hiszpanski and T. Y.-J. Han, *J. Chem. Inf. Model.*, 2020, **60**, 6147–6154.
